# Predicting peak inundation depths with a physics informed machine learning model

**DOI:** 10.1038/s41598-024-65570-8

**Published:** 2024-06-27

**Authors:** Cheng-Chun Lee, Lipai Huang, Federico Antolini, Matthew Garcia, Andrew Juan, Samuel D. Brody, Ali Mostafavi

**Affiliations:** 1 Urban Resilience.AI Lab, Zachry Department of Civil and Environmental Engineering, Texas A&M University, College Station, TX USA; 2 Institute for a Disaster Resilient Texas, Texas A&M University, College Station, TX USA; 3 Civil and Environmental Engineering, Rice University, Houston, TX USA

**Keywords:** Flood depth forecast, Machine learning, Interpretable model, Near-time prediction, Hydrology, Natural hazards

## Abstract

Timely, accurate, and reliable information is essential for decision-makers, emergency managers, and infrastructure operators during flood events. This study demonstrates that a proposed machine learning model, *MaxFloodCast*, trained on physics-based hydrodynamic simulations in Harris County, offers efficient and interpretable flood inundation depth predictions. Achieving an average $R^2$ of 0.949 and a Root Mean Square Error of 0.61 ft (0.19 m) on unseen data, it proves reliable in forecasting peak flood inundation depths. Validated against Hurricane Harvey and Tropical Storm Imelda, *MaxFloodCast* shows the potential in supporting near-time floodplain management and emergency operations. The model’s interpretability aids decision-makers in offering critical information to inform flood mitigation strategies, to prioritize areas with critical facilities and to examine how rainfall in other watersheds influences flood exposure in one area. The *MaxFloodCast* model enables accurate and interpretable inundation depth predictions while significantly reducing computational time, thereby supporting emergency response efforts and flood risk management more effectively.

## Introduction

As flood occurrences become more intense and frequent, decision makers, emergency managers, infrastructure owners and operators, and first responders have struggled to expeditiously assess and respond to flood accidents. This is in part due to a lack of timely, accurate, and reliable information on complex developed systems. Typically, physics-based hydrodynamic (i.e., hydrologic and hydraulic) models are used to compute flood hazards at particular areas of interest. However, these models could get computationally expensive and, worse, prohibitive, especially with increasing scale (both spatial and temporal), level of detail (resolution), and complexity (e.g., presence of hydraulic structures and multiple flood drivers). Minimizing the computational cost while maintaining the model’s accuracy, reliability, and interpretability is therefore paramount for near-time flood inundation estimation and prediction. Earlier studies had attempted to reduce the computational burden in flood warning systems by relying on real-time precipitation as an indicator of flooding potential^1–4^ or as a trigger to estimate inundation extent from a library of pre-delineated flood hazard maps^5,6^. Alternatively, geometric methods based on terrain topography, such as height above nearest drainage (HAND)^7^, showed promising results for short runtime flood prediction when coupled to reduced-physics models^8,9^. Despite these efforts, the flood hazard information provided remains limited in either scope (e.g., only considering the riverine floodplain) or scale (e.g., watershed or sub-watershed extent) due to various simplifying assumptions made in these systems. In an effort to take advantage of forecast product (2–15 days) within a hydrodynamic model framework considering multiple flood sources, Wing et al.^10^ combined the extraction of fluvial flooding from a library of pre-modeled flood maps and pluvial and coastal surge flooding resulting from an expedite, coarse-resolution physics-based model. While the results show that hydrodynamic models are more accurate than non-physics ones, it remains unclear if the former can be adapted for same-day forecast and for flood events of any intensity and spatial pattern. In addition, existing models have limited interpretability to decode the extent to which different features shape flood exposure. Such interpretability in examining the importance of different features is essential for characterizing regional flood exposure and formulating flood mitigation measures.

In recent years, machine learning (ML) has emerged as a powerful tool in flood inundation predictions^11–13^, with numerous studies utilizing it to assess various aspects of floods, including flood damage, flood disruptions, and other relevant applications. Specifically, Hou et al.^14^ employed a combination of machine learning algorithms, including Random Forest and K-Nearest Neighbor, alongside a hydrodynamic-based urban flood model to predict inundation area, depth, and volumes. Similarly, Motta et al.^15^ developed a flood prediction system that integrated machine learning classifiers and GIS techniques to identify flood-prone areas. However, a limitation of their work is the lack of determination of feature contribution to prediction, which is critical for interpreting model outputs accurately. Addressing this important aspect, Zahura and Goodall^16^ utilized Random Forest to create a surrogate model trained on environmental features from various flood events, predicting flood extent and depth in an urban coastal watershed while exploring the contribution of different physical features to localized flooding. However, a significant gap in their study is the lack of consideration for interactions from nearby and upstream areas, which is crucial in achieving accurate flood prediction. To address this gap, some researchers have explored the use of deep learning and spatial reduction and reconstruction methods^17,18^. Nonetheless, while these complex models may offer improved predictive capabilities, they often come at the cost of reduced interpretability. Recent studies^19,20^ have shown the potential of using interpretable deep learning models for evaluating feature importance in flood nowcasting. However, these studies are limited in evaluating flood status in neighborhoods as a binary state (flooded or non-flooded) and do not enable predicting peak flood depth which is essential information for evaluating the extent of risk and expected damage.

In light of these challenges and advancements in the field, this paper introduces *MaxFloodCast*, a surrogate machine learning modeling framework with novel feature engineering. *MaxFloodCast* provides peak inundation predictions while taking into account nearby and upstream precipitation information, all while maintaining model interpretability. The *MaxFloodCast* model is trained and tested using physics-based model simulations to address concerns regarding the scarcity of historical inundation data^21^. We applied *MaxFloodCast* in the multi-watershed region of Harris County (Texas, USA), and validated it by reproducing 2017 Hurricane Harvey and 2019 Tropical Storm Imelda events. *MaxFloodCast*’s predicted peak flood levels compared with a physics-based model (HEC-RAS 2D).

Surrogate models’ ability to generate results qualitatively similar to standalone hydrodynamic models at a fraction of time opens the way to various applications, including regional floodplain management and real-time emergency operations. Specifically, *MaxFloodCast* can have a significant impact on two aspects: (1) the reduced computational cost and expedite prediction facilitate timely and effective emergency response, for example by identifying inundated roads and disrupted access to facilities, and provide early insights to public officials for property damage estimation; (2) the evaluation of features that determine inundation extent in different areas offers insights to flood managers and urban planners into flood mitigation strategies that would reduce local and regional peak inundation depths in future events. By integrating the strengths of machine learning and physics-based models, *MaxFloodCast* model could offer an efficient approach to flood peak inundation prediction and feature evaluation, enabling more effective floodplain management and informed decision-making on flood risk mitigation.

## Results

**Figure 1:**
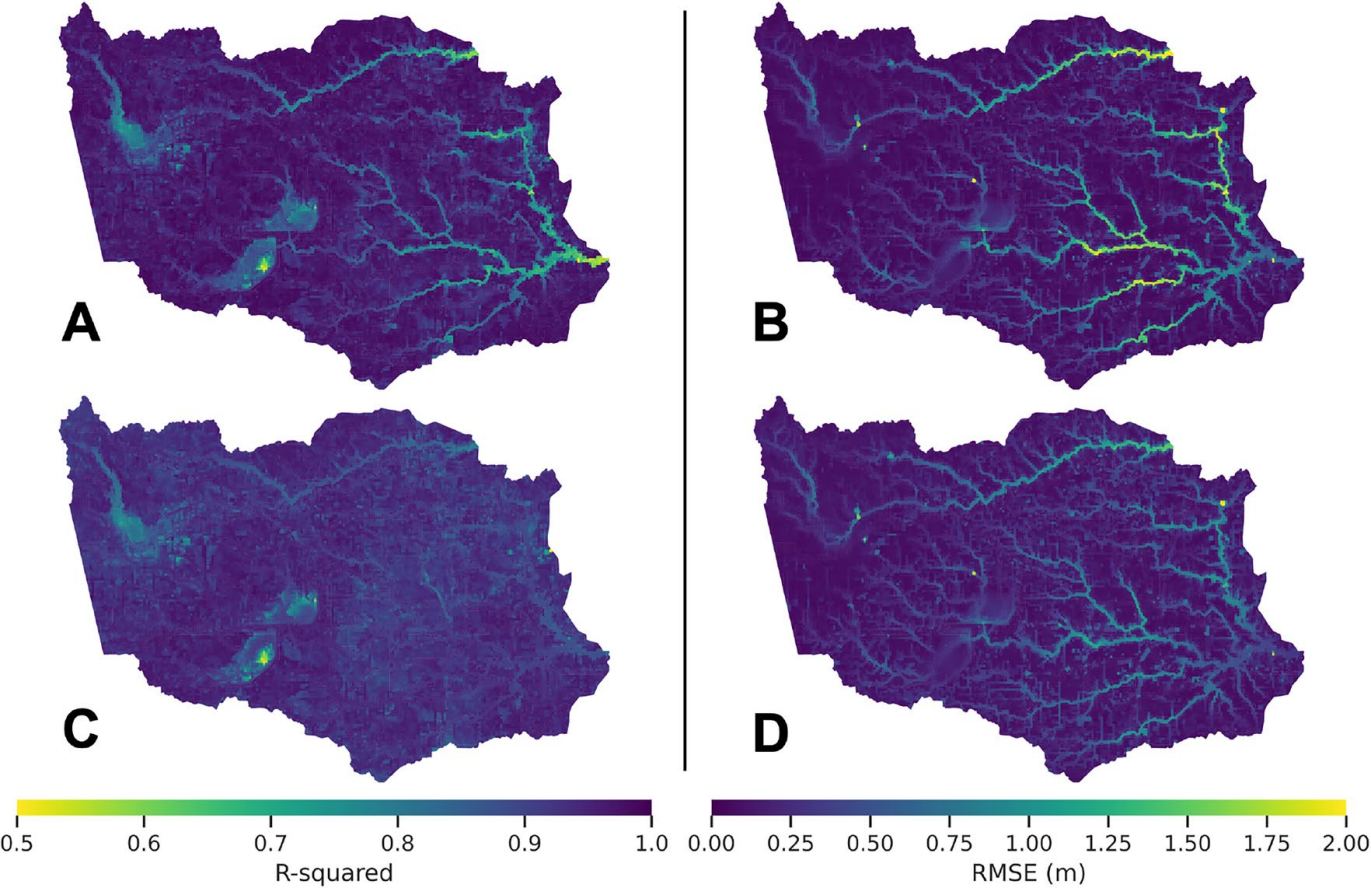
Test $R^2$ and RMSE of Experiments 1 (A and B) and 2 (C and D). Maps generated with *geopandas v0.14.3* (https://geopandas.org/).

The study domain includes the majority of Harris County, Texas (USA), represented by 26,301 polygonal cells (details provided in Section "Study area"). The cells are further classified between “channel” and “non-channel” cells. Channel cells represent major streams (natural or manmade), while non-channel cells represent the overland areas outside of water bodies, which could drain into nearby channel cells or be disconnected from them entirely. Since the dominating hydrologic processes occurring on a channel cell differ from those of a non-channel cell, we created and trained an individual machine-learning model for each cell. The analysis includes two experiment setups utilizing tree-based XGBoost method. The first experiment setup (Experiment 1) considers two precipitation features: hourly intensity peak and cumulative precipitation, for a given storm event, within each cell. In the second setup (Experiment 2), we added two features, heavy peak precipitation ratio and heavy cumulative precipitation ratio, which incorporate watershed-wise information about rainfall spatial distribution. The specific details of these two features are discussed in Section "Precipitation data and corresponding feature engineering". A total of 592 simulated synthetic storm events with varying rainfall intensity, magnitude, and duration (details provided in Section "Physics-based model setup and ground truth data") were divided into 60% for model training, 20% for validation, and 20% for testing purposes. We assessed *MaxFloodCast* performance by calculating the correlation ($R^2$) and the average distance (Root Mean Squared Error, RMSE) between the ML-predicted maximum water depths for each cell and the corresponding maximum depths resulting from a physics-based model (HEC-RAS 2D, see Section "Physics-based model setup and ground truth data" for details) for the same storm event, for all the events in the testing set.The test $R^2$ and RMSE of Experiments 1 and 2 are presented in Table 1 and Fig. 1.

**Table 1:** Average test $R^2$ and Root Mean Squared Error values of *MaxFloodCast* Experiments 1 and 2.

	Experiment 1			Experiment 2		
	Channel	Non-channel	Overall	Channel	Non-channel	Overall
R$^{2}$	0.878	0.953	0.944	0.916	0.928	0.926
RMSE	2.17 ft (0.66 m)	0.46 ft (0.14 m)	0.66 ft (0.20 m)	1.76 ft (0.54 m)	0.57 ft (0.17 m)	0.71 ft (0.22 m)

Experiment 1 demonstrates strong performance in predicting inundation depth in the non-channel cells using only peak and cumulative precipitation within each cell, with an $R^2$ value of 0.953 and an RMSE of 0.46 ft (0.14 m). However, the performance in the channel cells is comparatively weaker, likely due to Experiment 1 setup not considering precipitation in contributing nearby and upstream drainage areas. In Experiment 2, including precipitation information from contributing watersheds and drainage areas led to improved results in the channel cells, with an $R^2$ value increasing from 0.878 to 0.916 and an RMSE decreasing from 2.17 ft (0.66 m) to 1.76 ft (0.54 m). Nonetheless, while beneficial for improving the prediction of the ML models in channel cells, watershed-wise features slightly worsen the prediction of ML models in non-channel cells, likely due to the introduction of noise and extraneous information, leading to overfitting and increased model complexity. Considering that approximately 80% of the cells are non-channel cells, the overall performance of Experiment 2 is inferior to that of Experiment 1.

### Differences between channel and non-channel cells

**Figure 2:**
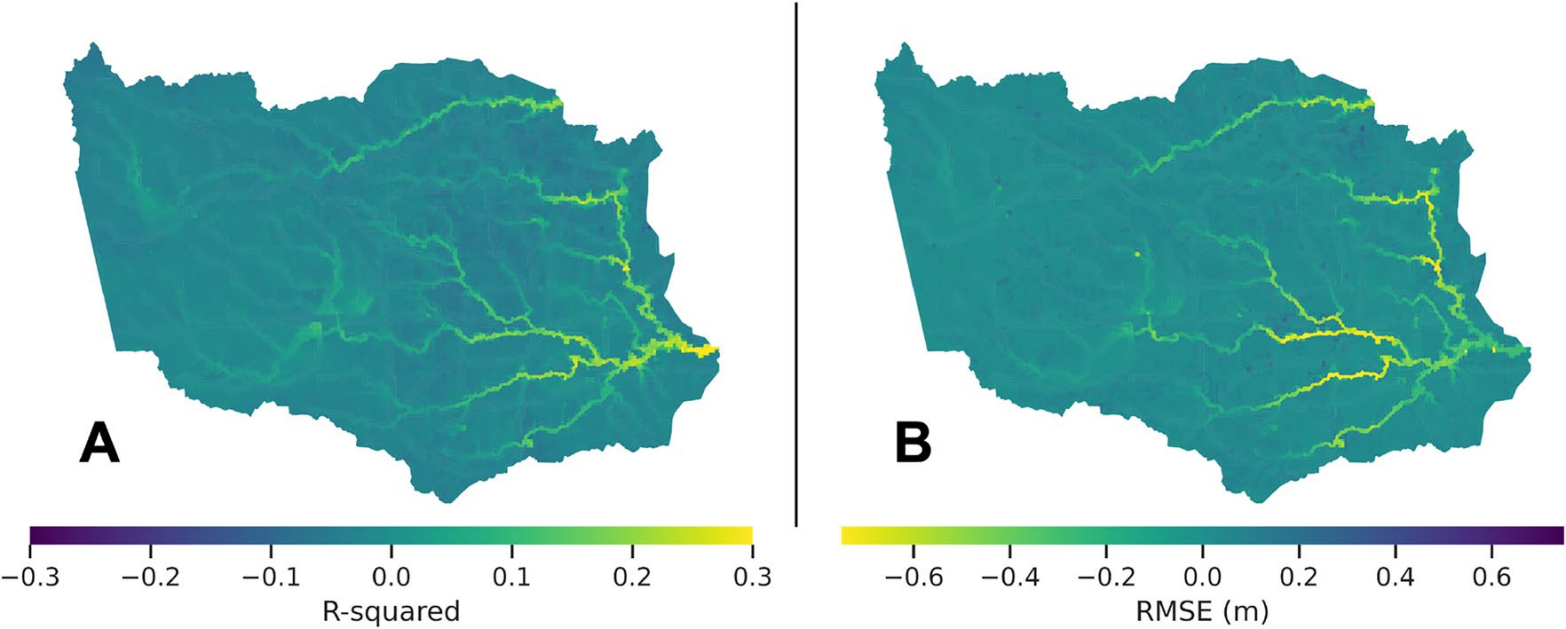
Differences in $R^2$ (**A**) and RMSE (**B**) between Experiments 1 and 2, with yellow indicating improvement. Maps generated with *geopandas v0.14.3* (https://geopandas.org/).

Based on the results, Experiment 2 demonstrates improvements in both $R^2$ and RMSE values for the channel cells but worsens the performance in the non-channel cells. Fig. 2 illustrates the differences in $R^2$ and RMSE between Experiments 1 and 2, with yellow indicating improvement. As depicted in Fig. 2, a significant number of channel cells have an increase of more than 0.2 in $R^2$ and a decrease of more than 1 ft (0.31 m) in RMSE.

These findings indicate that considering precipitation in nearby and upstream contributing drainage areas improves the prediction performance of the ML models for the channel cells. However, the inclusion of additional features results in decreased performance of the ML models in the non-channel cells. Because each of the 26,301 ML models is independent from the others, we considered ML models with Experiment 1 setup on non-channel cells and ML models with Experiment 2 setup on channel cells, and evaluated the overall prediction capability of *MaxFloodCast*. As shown in Table 1, the results indicate that the combined performance of Experiment 1 and Experiment 2 can reach an $R^2$ of 0.949 and an RMSE of 0.61 ft (0.19 m). These results show the capability of the *MaxFloodCast* model in producing accurate and reliable peak inundation predictions in both channel and non-channel cells.

### Feature importance in the prediction of peak inundation depth

**Figure 3:**
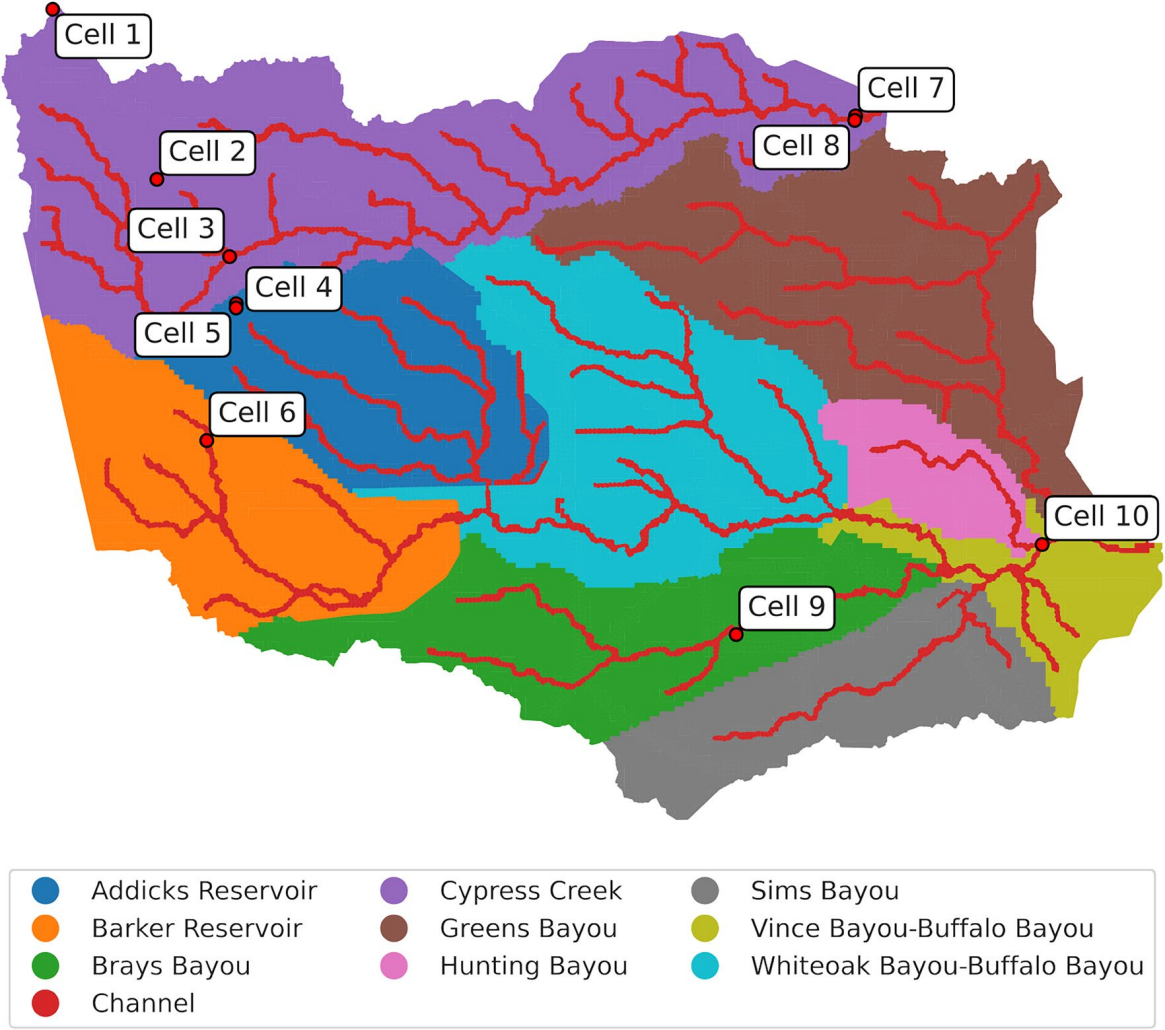
Locations of the 10 selected cells chosen for further discussion. Map generated with *geopandas v0.14.3* (https://geopandas.org/).

*MaxFloodCast* results are hereby interpreted by assessing weighted features and their alignment with engineering judgment. Experiment 2 incorporates 21 features (details in Section "Physics-based model setup and ground truth data"), representing the precipitation status in various areas within Harris County for model training. We selected 10 cells in the study area representing different contexts and characteristics, including upstream/downstream and channel/non-channel cells, for further analysis. The locations and descriptions of these 10 selected cells are provided in Fig. 3 and Table 2, respectively.

**Table 2:** Details of the 10 selected cells chosen for further discussion.

Cell	Location description	Watershed
1	Study area perimeter	Cypress Creek
2	Area disconnected from the channel network	Cypress Creek
3	Cell containing two distinct channels differing by size, drainage, and streambed elevation	Cypress Creek
4	Area on the edge of a steep terrain depression	Addicks Reservoir
5	Terrain depression (quarry)	Addicks Reservoir
6	Upstream channel	Barker Reservoir
7	Downstream channel	Cypress Creek
8	Downstream floodplain	Cypress Creek
9	Residential area in the floodplain	Brays Bayou
10	Downstream channel, after a major confluence	Buffalo and Hunting Bayous

**Figure 4:**
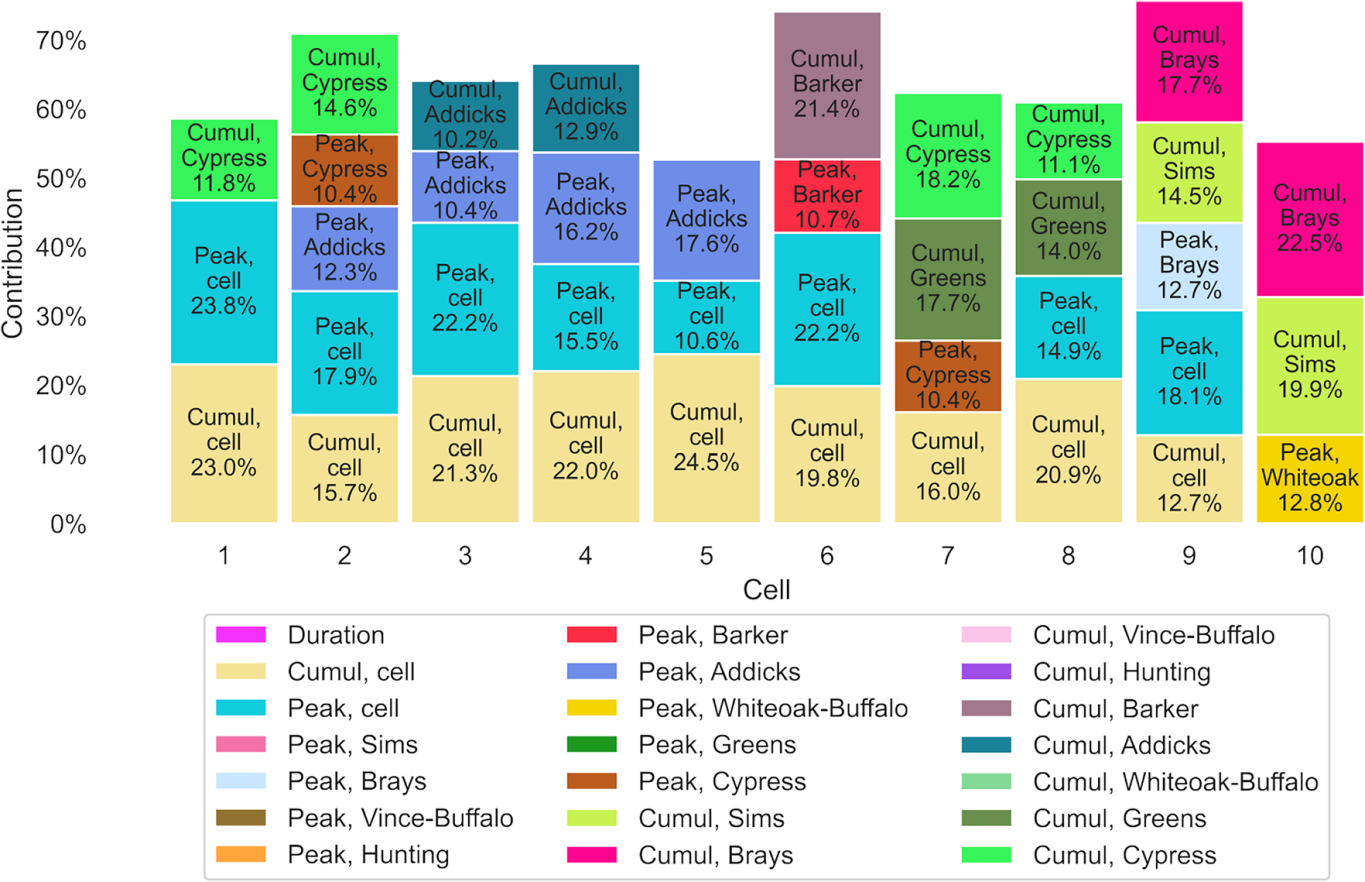
Features that contribute more than 10% to the depth prediction for the 10 selected cells (*Note*: ‘Cumul’ and ‘Peak’ followed by a watershed name are to be intended as heavy cumulative precipitation ratio and heavy peak precipitation ratio for that watershed, as defined in Section "Precipitation data and corresponding feature engineering").

The *MaxFloodCast* model is based on XGBoost method. One of the key benefits of using XGBoost is its ability to identify and prioritize essential features, consequently enhancing both model accuracy and interpretability. XGBoost provides valuable insights into each feature’s contributions, enabling a better understanding of their importance in making predictions. Fig. 4 demonstrates the features that contribute more than 10% to the prediction of inundation depth for the 10 selected cells.

The cumulative and peak precipitations within the cells play a primary role in predicting inundation depth for most of the selected cells, except for cells 7 and 10, which are both downstream channel cells. Water depth prediction in cell 7 is affected by the heavy cumulative precipitation ratio and heavy peak precipitation ratio in its watershed (Cypress Creek) and by the heavy cumulative precipitation ratio in a nearby watershed (Greens Bayou). More notably, for cell 10, which has the largest drainage of the selected cells, both cell peak and cell cumulative precipitation contribute less than 10% to its depth prediction. Instead, depth prediction for cell 10 is predominantly influenced by its upstream watersheds, specifically the heavy cumulative precipitation ratio in Brays Bayou and Sims Bayou watersheds, as well as the heavy peak precipitation ratio in the Whiteoak Bayou-Buffalo Bayou watershed.

In general, depth peak at high-drainage cells, such as channel cells in midstream and downstream sections of a watercourse, is mostly determined by the water received from upstream rather than direct rainfall. This explains why depth peak on high-drainage cells was poorly predicted in Experiment 1, where no information on upstream precipitation was used to train the models. The same prediction improved and was significantly influenced by watershed-wide precipitation features in Experiment 2. For instance, peak prediction on floodplain cell 8 relies more on precipitation information within its cell, whereas the prediction on its high-drainage neighbor, the abovementioned channel cell 7, is mostly determined by larger scale features (Cypress Creek peak, Cypress Creek cumulative and Greens Bayou cumulative).

The findings of this study demonstrate that the trained ML models can better predict inundation depth by considering features that align with the physical characteristics of the study area as well as engineering judgment. The downstream cells show a tendency to rely on upstream information, whereas predicting inundation in channel cells exhibits a greater need for information from both nearby and upstream areas compared to non-channel cells. At a more general level, these results reveal the necessity to train ML models using features defined at a scale larger than a single cell, to account for the spatial nature of overland flow, and also to smooth the uncertainties related to the spatial and temporal resolutions of precipitation grids.

### Model validation with Hurricane Harvey and Tropical Storm Imelda

To validate the effectiveness of the developed ML model in predicting flood inundation depth in Harris County, we utilized two severe storm events, Hurricane Harvey and Tropical Storm Imelda, as case studies in the study area. Hurricane Harvey, occurred in August 2017 and stands as one of the most devastating storms that caused catastrophic flooding in Texas, impacting the Texas Gulf Coast and resulting in extensive damage to properties and infrastructure. On the other hand, Tropical Storm Imelda struck the Houston metropolitan area in September 2019. While not as intense as Harvey, Imelda still caused widespread flooding in several regions, affecting homes, businesses, and transportation systems. These storms were selected for their contrasting characteristics, including size, rainfall distributions, and total rainfalls, allowing the ML models to be validated on larger and more generally uniform rainfall events, such as Harvey, as well as more localized and scattered events, like Imelda. This paper considers peak water depth records collected at channel gages (see Section "Physics-based model setup and ground truth data" for more details) during Hurricane Harvey and Tropical Storm Imelda to validate the peak depth prediction of the surrogate ML models. The performance of the physics-based model, HEC-RAS 2D is also provided for reference. HEC-RAS 2D (described in Section "Physics-based model setup and ground truth data") simulated 168 hour-long (7 days) Harvey and Imelda using a time step of 5 minutes. Tables 3 and 4 present the Root Mean Squared Error (RMSE) and Mean Absolute Percentage Error (MAPE), calculated from gage peak values, as performance metrics for the three models. Finally, Tables 3 and 4 list computational time for each simulation on a mid-range laptop (*12th Gen Intel(R) Core(TM) i7-1270P at 2.20 GHz, 32GB RAM, Windows 10 Enterprise*).

**Table 3:** Model runtime and validation of peak depths versus peak gage records for Hurricane Harvey.

	Gage depth < 15ft (4.57m): (n=24)		Gage depth $\ge$ 15ft (4.57m) and < 25ft (7.62m): (n=40)		Gage depth $\ge$ 25ft (7.62m): (n=34)		Runtime
	RMSE	MAPE	RMSE	MAPE	RMSE	MAPE	
HEC-RAS 2D	3.24 ft (0.99 m)	14.66%	2.38 ft (0.73 m)	8.48%	3.48 ft (1.06 m)	8.93%	3m 30s
ML-Exp1	2.21 ft (0.67 m)	13.28%	3.66 ft (1.12 m)	13.96%	7.25 ft (2.21 m)	17.68%	1m 32s
ML-Exp2	2.50 ft (0.76 m)	15.22%	4.06 ft (1.24 m)	16.20%	7.62 ft (2.32 m)	18.50%	1m 46s

**Table 4:** Model runtime and validation of peak depths versus peak gage records for Tropical Storm Imelda.

	Gage depth < 8ft(2.44m): (n=23)		Gage depth $>=$ 8ft(2.44m) and < 15ft(4.57m): (n=40)		Gage depth $>=$ 15ft(4.57m): (n=37)		Runtime
	RMSE	MAPE	RMSE	MAPE	RMSE	MAPE	
HEC-RAS 2D	4.83 ft (1.47 m)	236.16%	3.59 ft (1.09 m)	23.37%	4.46 ft (1.36 m)	15.72%	2m49s
ML-Exp1	4.15 ft (1.27 m)	126.11%	3.68 ft (1.12 m)	27.3%	5.34 ft (1.63 m)	20.84%	1m36s
ML-Exp2	3.77 ft (1.15 m)	158.23%	3.26 ft (0.99 m)	24.09%	5.35 ft (1.63 m)	20.01%	1m47s

Hurricane Harvey had larger and more uniform rainfall, with almost the entire Harris County experiencing precipitation heavier than two inches, the threshold used to define heavy precipitations ratios in this study. These features, introduced in Experiment 2, do not lead to an improvement in prediction performance. In fact, these additional features seem to weaken the model by potentially introducing noise and extra information. Specifically, when the gage depth is less than 15 ft (4.57 m), the ML models perform similarly to the physics-based model in terms of MAPE and even outperform it in terms of RMSE. However, as the gage depth exceeds 15 ft (4.57 m), the performance of the ML models declines. This could be attributed to the lack of events of the same magnitude as Hurricane Harvey in the training data, resulting in a worse prediction performance. For reference, our findings are along the lines of^10^ reporting a RMSE between 1.71 m and 1.88 m when comparing results from their forecast model and USGS high-water marks for Hurricane Harvey. Model performances are not comparable due to different study domains and different definitions of accuracy and RMSE.

On the other hand, for Tropical Storm Imelda, which had non-uniform, less intense, and shorter duration rainfalls compared to Hurricane Harvey, the additional precipitation features in Experiment 2 slightly improve the prediction performance compared to Experiment 1. This result highlights the value of incorporating spatially relevant precipitation information when rainfall distribution is not uniform. Furthermore, the results demonstrate that the prediction performance of the ML models during Tropical Storm Imelda is comparable to the physics-based model when the gage depth is less than 15 ft (4.57 m) and only slightly worse when the depth exceeds 15 ft (4.57 m).

## Discussion

The study demonstrates that ML models, trained using physics-based model simulations, can provide comparable prediction performance to hydrodynamic models while improving computational efficiency and model interpretability. The *MaxFloodCast* model presented in this study provides a prediction performance aligned with the performance of a standalone hydrodynamic model in predicting peak flood depths for most of flood intensities considered. However, the prediction of extreme events is less successful, especially where flood depths are greater than 15 ft (4.57m, Tables 3 and 4). This was partially expected due to the lack of extreme events in the training set, and highlights the importance of training a model using many events covering the largest range of precipitation intensity, duration, and spatial pattern. Further, it is worth mentioning that storms producing large and widespread runoff trigger flow regulation operations by man-managed hydraulic structures, such as Addicks and Barker reservoirs in the case of Harris County. These alterations to the natural flow were not captured by *MaxFloodCast* for Harvey and Imelda events, and partially explain inconsistent predictions upstream and downstream those structures. Nonetheless, an implementation in the ML training of threshold-based instructions for hydraulic structure operations may not be necessary, since the purpose of the ML model is to provide decision-makers and operators a picture of where and how flood would occur, before eventual human intervention. Future work should improve on the promising findings presented in this paper and enhance *MaxFloodCast* potential to serve as a surrogate model to the physics-based model for the largest variety of cases, including extreme precipitation events.

In its current form, the ML presented in this paper only looks at peak flood depth as its output feature. Flood level represents a proxy for the severity of a flood event and is used in most damage functions to estimate flood damage to buildings and infrastructures. However, other features are also important for flood assessment. Time-to-peak is essential for timely decisions and intervention by emergency operators, in case of pluvial or fluvial floods. Inundation duration, often related to peak water stage, affects the length of emergency and recovery phases, and is also used in building damage modeling. Peak stage, time-to-peak and duration fully characterize a stage hydrograph. Eventually, the main objective of a ML model, as of any flood model, including physics-based ones, is to predict the hydrograph at every location over a study area. Future developments will aim at including more information in the training phase, and producing other hydrograph elements in addition to flood peak, to enhance the predictive capability of the ML model.

Simulation runtimes tend to increase with the model’s computation complexity, extent of modeled area, spatial and temporal resolutions, number and type of output generated, and duration of the simulated event. To minimize computing resources, scalability is an essential attribute of a model. Because *MaxFloodCast* consists of independent ML models, one for each cell, its runtime increases linearly with the number of cells/models. On the other hand, the computational time of HEC-RAS 2D, like other fully distributed models, increases with domain extent or 2D mesh spatial resolution more rapidly than linearly^22,23^. In comparison, *MaxFloodCast* is scalable, because as the study area extent increases, e.g., for regional studies, its runtime only increases linearly, while to perform the analysis with a physics-based model it may be necessary to decrease spatial and temporal resolution. *MaxFloodCast* is also flexible, because it is possible to limit flood prediction to a subset of contiguous cells, e.g., a watershed or a channel and the nearby floodplain, whereas a hydrodynamic model simulates all the physical processes occurring in the domain, on all channels and from source to outlet. Finally, *MaxFloodCast* is built to be computationally efficient. While runtimes in Tables 3 and 4 do not show a large difference between the two models, *MaxFloodCast* is conceived to be parallelized on a multi-core machine or a high-performance computing infrastructure, with a drastic abatement of computational cost, whereas several challenges exist when adapting physics-based models for parallelization^24^.

The interpretability and evaluation of watershed interdependencies are unique aspects that set *MaxFloodCast* model apart from existing flood prediction models. The feature importance analysis offers critical information to examine how flood exposure in one place is influenced by the rainfall on that very place, on the related drainage area, and on nearby watersheds. These insights could enable a more system-of-systems approach to flood risk assessment. Understanding what features lead to frequent and/or severe inundation of a place allows one to discover what flood dynamics (pluvial, bank overtopping, backwater effect) are prevalent and what events trigger those dynamics. For example, on an elongated watershed, where a flood wave on the main channel attenuates along its course, flash flooding on a downstream cell may be explained by the precipitation pattern observed on neighboring catchments, beside the precipitation on that cell and its drainage area. Incorporating features that describe rainfall patterns at different spatial scales is a key development for *MaxFloodCast*, and for all ML models that mean to bypass the physical simulation of hydrologic and hydraulic processes. Furthermore, the model’s ability to be re-trained over time, based on the updated data from hydrodynamic modeling results that consider city development and flood mitigation measures, enhances its practicality for future events.

In sum, the integration of physics-based simulations and ensemble machine learning techniques, particularly XGBoost, presents a promising framework for near-time flood prediction and management. The proposed *MaxFloodCast* model takes rainfall data as input and produces peak inundation predictions for future events. Having computationally faster and reliable predictions of maximum flood inundation depth is crucial for flood managers, emergency managers, and decision-makers planning and responding to flood events. By compressing the planning-response cycle, losses from flooding in vulnerable areas could be more effectively reduced. The model is adaptable to other regions in the U.S. and across the world, given proper training on location-specific physics-based models.

## Materials and methods

### Study area

**Figure 5:**
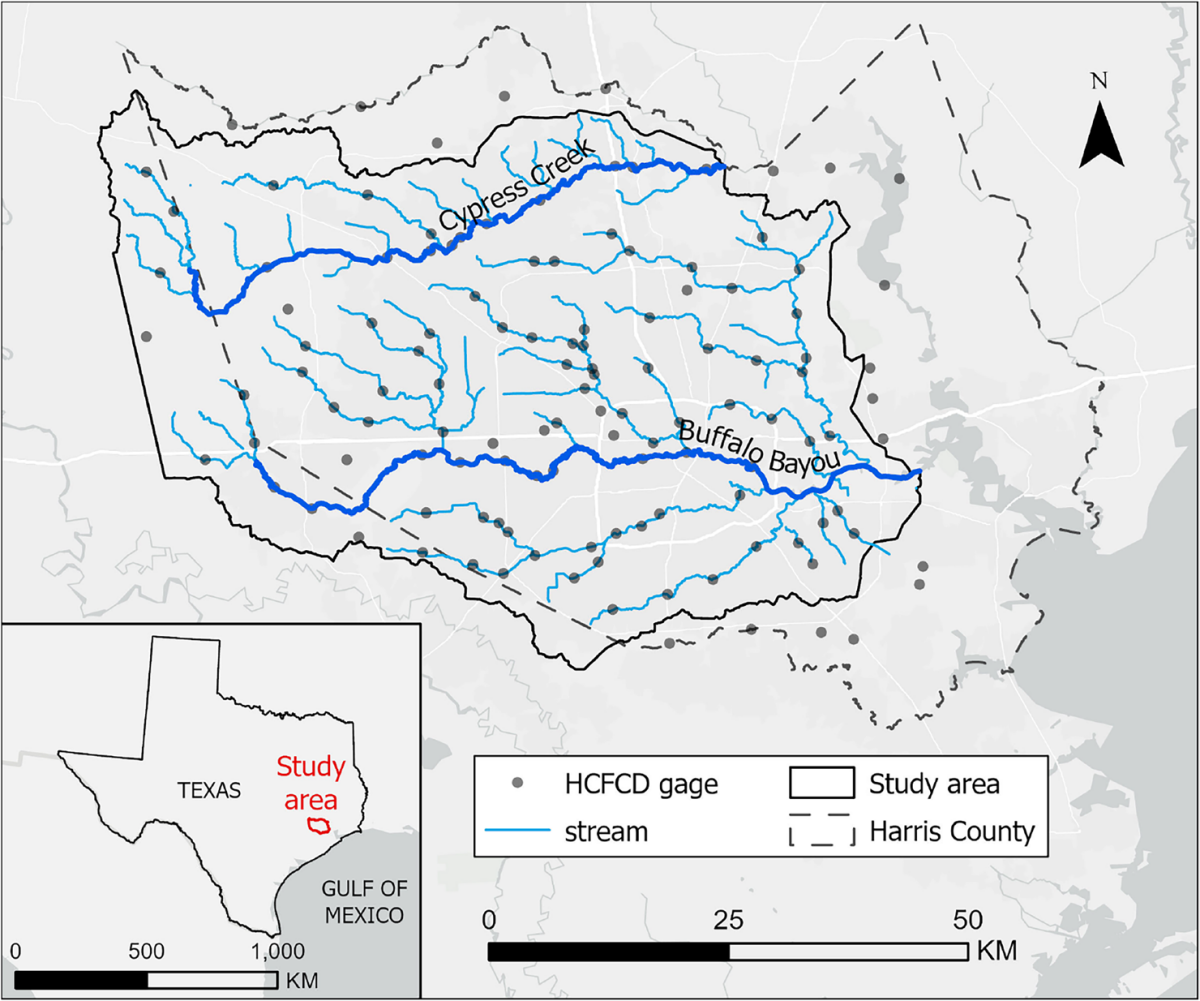
Study area and Harris County Flood Control District gage distribution. Map created with ArcGIS Pro 3.0.0 (https://pro.arcgis.com/).

Harris County, in Southeast Texas, is the largest county of the Greater Houston Metropolitan Statistical Area, with an area of 1,778 sq mi (4,605 sq km) and a population that grew past 4.5 million people in the past ten years. The topography is substantially flat, with elevation ranging from -40 ft (-12.19 m) up to 300 ft (91.44 m) above mean sea level in the northwest corner. More than two-thirds of the county area is classified as developed, while about 20% is classified as pasture and cultivated in the western part of the county^25^. The two main watersheds are Cypress Creek in the north and the Buffalo Bayou system in the central and southern portions of the county. Both flow eastward into the low course of the San Jacinto River and the Ship Channel, which eventually are connected to the Gulf of Mexico. The presence of a densely built environment, slow natural drainage, and scarce soil infiltration capacity, together with its location in a subtropical climatic region, make Harris County chronically prone to flooding. Fig. 5 shows the details of the study area along with the boundary of Harris County.

### Physics-based model setup and ground truth data

*MaxFloodCast* is trained using physics-based simulations of 592 synthetic storm events to address the challenges arising from the scarcity of historical inundation data. Synthetic storms were obtained applying Rasterized Time-series Resampling Method to historic storms in the area, as illustrated in^26^. Storms vary by duration (from 1 up to 33 hours), precipitation intensity and spatial movement across the study area, and are stored in Network Common Data Form (NetCDF) files, with hourly grids having a resolution of 3,315 ft (1,010 m). Simulated flood depths are provided by the HEC-RAS 2D model, which is a hydrologic software developed by the Army Corps of Engineers. In this study, we built a two-dimensional model for unsteady flow simulation. The study area was covered with a 1,200 ft x 1,200 ft (approximately 366 m) mesh grid, which was further refined along the major watercourses and tributaries with computations along these centerlines to form pseudo-cross sections for stability and computational speed. Breaklines were applied in correspondence to interstates, highways, and other known high points to improve accuracy. The resulting mesh comprises 26,301 cells, providing a detailed representation of the study area. To establish the necessary parameters for the simulation, we implemented Manning’s roughness and imperviousness values based on the 2019 National Land Cover Dataset^25^. Additionally, soil infiltration parameters were acquired from the Gridded Soil Survey Geographic (gSSURGO) database^27^.

To validate the HEC-RAS 2D model, all Harris County Flood Control District gages^28^ were analyzed for completeness of data during five events spanning from 2016 to 2020. These events spanned a wide range of types, from extremely localized, like the 2016 Tax Day event, to widely uniform, like the 2017 Hurricane Harvey event. These concluded with Tropical Storm Beta in 2020, as this is when the original model was developed. The gages and events were then calibrated for global minimum error on all gages since there were significant deviations on flow runoff volumes from different parts of the domain with similar land use and soil type from antecedent moisture conditions and other similar factors that were all globally set for each event. This model was developed for a methodology paper showing that it was worth pursuing ML for these types of large computationally heavy Partial Differential Equations solvers for surrogate modeling^29^.

Besides HEC-RAS 2D model results, this study looks at data from 2017 Hurricane Harvey and 2019 Tropical Storm Imelda to validate the ML model. We leveraged Harris County Flood Control District’s network of rain and stream gages^28^, which makes Houston one of the most gaged cities in the country^30^. We used rain gage records from 122 stations to model the rainfall^28^. These gages have a high temporal resolution, with records every 15 minutes, and are well-distributed within and outside the study area (Fig. 4.1), making them more reliable than other data sources for the use case. We considered high-water mark records collected by USGS after Hurricane Harvey^31^, which would have worked as validation points outside of gauged channels. However, high-water marks and gage records showed strong inconsistencies, with differences up to thirty feet on the same point for some locations, and we preferred to rely on stream gages only. Future work will look at damage records to attempt estimating water height at property level outside of channels.

We modeled 7-day periods, 8/25-9/1/2017 for Hurricane Harvey and 9/15-9/22/2019 for Tropical Storm Imelda, in HEC-RAS 2D using the Thiessen polygons method. We then extracted peak hourly intensity, cumulative precipitation, and event duration values at the cell level, which feed the ML model. Later, we compared *MaxFloodCast* peak depths against gage peak depths. To compare depths from the same baseline, we calculated gage depths by subtracting water surface elevation recorded or reconstructed at the gage stations from the terrain elevation used in HEC-RAS 2D.

### Precipitation data and corresponding feature engineering

When feeding input rainfall to the ML model, we interpolated the rain grids from each of the 592 storm events to estimate the hourly rainfall within each model cell. This approach treats the precipitation of each cell as a time series, from which cumulative and peak values are extracted.

**Figure 6:**
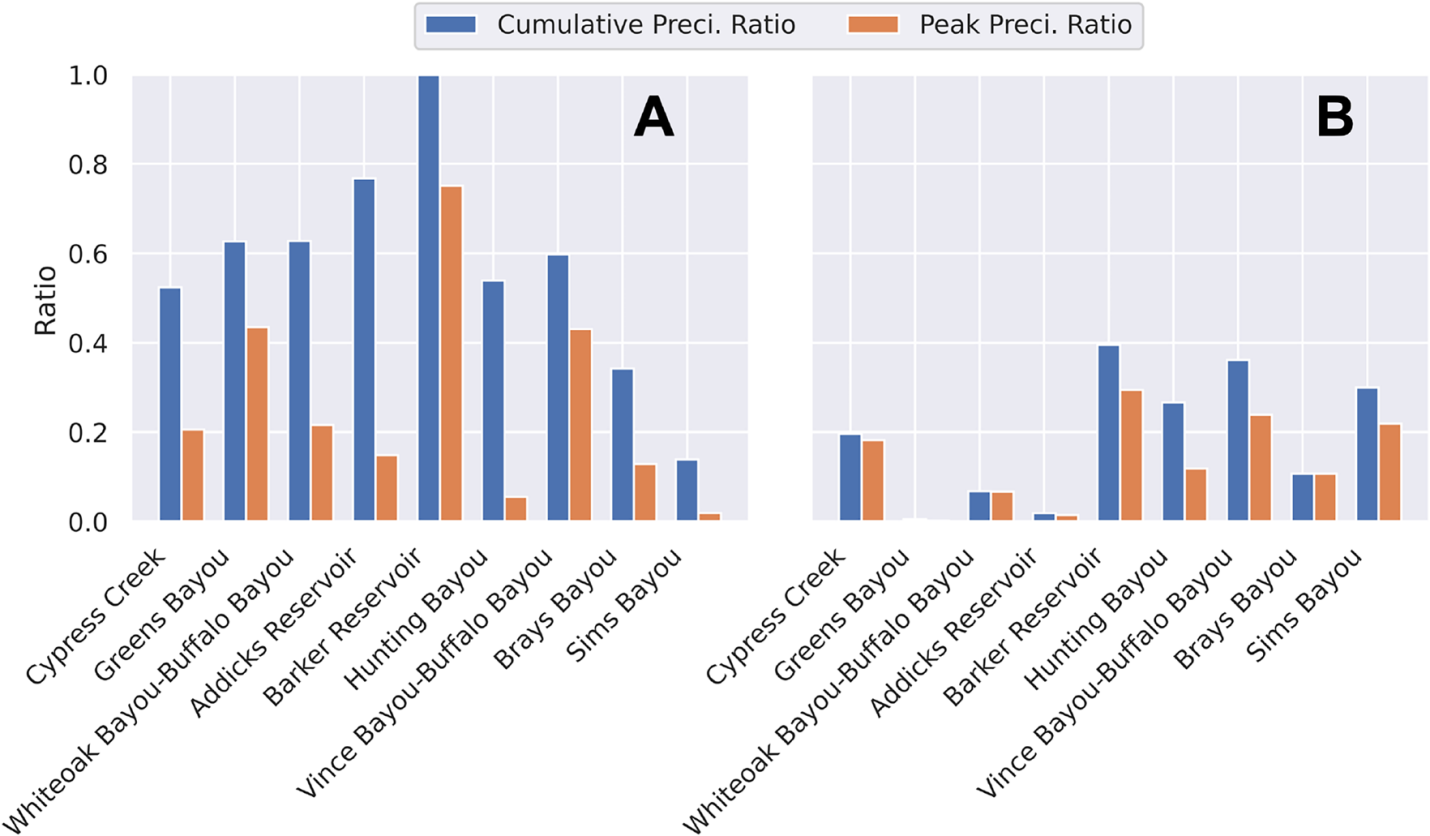
Heavy cumulative and heavy peak precipitation ratios of two storm events used for *MaxFloodCast* training. Event (**A**) is a 6-h precipitation event, and Event (**B**) is a 2-h precipitation event. Their corresponding cumulative and peak rainfall maps are provided as Supplementary Information.

In addition to cell cumulative and peak precipitation data, we defined heavy peak precipitation ratio and heavy cumulative precipitation ratio at the watershed level to account for the spatial occurrence of intense precipitation in the study area (Equation 1 and 2). These intermediary features enhance the understanding of precipitation patterns in different areas, thereby improving the overall predictive modeling performance. We considered nine watershed regions in the study area, as depicted in Fig. 3. For each watershed region, the heavy peak precipitation ratio and the heavy cumulative precipitation ratio are calculated based on the area of the cells where heavy precipitation occurred. We define heavy precipitation based on a threshold of two inches (50.8 mm), which is the rain intensity for a 1-h event with a return period of at least two years in the study area. For a given precipitation event, a binary variable is assigned to each cell, with 1 indicating that the peak (cumulative) precipitation was above the threshold on the cell, 0 if the peak (cumulative) precipitation value was within the 2-inch threshold. The ratio is given by the sum of the areas of the cells marked with 1 divided by the area of the watershed. The following equations define heavy peak precipitation ratio and heavy cumulative precipitation ratio for a given watershed *i*:

1 $$\begin{aligned} \text {Heavy Peak (Cumulative) Precipitation Ratio}_i = \frac{\sum _{j}^{}AC_{i,j}\times h_{i,j}}{AW_{i}} \end{aligned}$$

2 $$\begin{aligned} h_{i, j} = {\left\{ \begin{array}{ll} 1, &{} \text {if } p_{i, j} > 2 \text { in}, \\ 0, &{} \text {otherwise}. \end{array}\right. } \end{aligned}$$

where $AW_i$ represents the area of watershed *i*, $AC_{i, j}$ represents the area of cell *j* in watershed *i*, $h_{i, j}$ is a binary identifier of cell *j* in watershed *i*, and $p_{i, j}$ denotes the peak (cumulative) precipitation in cell *j* within watershed *i*. Heavy peak precipitation ratio and heavy cumulative precipitation ratio are defined at the watershed level and thus are constant across all cells of a watershed for a given rainfall event. These two ratios can vary significantly across different events and watershed regions, providing substantial explanatory potential. For instance, as illustrated in Fig. 6A, a higher heavy cumulative precipitation ratio is not necessarily associated with a higher heavy peak precipitation ratio. On the other hand, Fig. 6B demonstrates that while the heavy cumulative precipitation ratio and heavy peak precipitation ratio might be correlated, their values in different watershed regions are distinct and represent how the precipitation pattern varies spatially. For example, the storm in Fig. 6B was short (similar ratios mean peak and cumulative values are also similar) and scattered, since it was more intense in the southern (Barker, Vince-Buffalo, Hunting and Sims) and western (Cypress) parts of the study area. These observations highlight the importance of considering both ratios to gain comprehensive insights into precipitation characteristics during various events and across diverse geographic areas. Table 5 lists all features utilized in this paper for training the ML model to predict flood inundation depth.

**Table 5:** Features utilized in this study for training ML models cell-wise.

Feature	Unit	Description
Cumulative precipitation	Inch	Total precipitation
Peak precipitation	In/hour	Maximum precipitation intensity
Duration	Hour	Precipitation duration
Nine heavy cumulative precipitation ratio (one for each watershed)	Scalar (range 0 to 1)	Proportion of watershed area experiencing heavy cumulative precipitation
Nine heavy peak precipitation ratio (one for each watershed)	Scalar (range 0 to 1)	Proportion of watershed area experiencing heavy peak precipitation

### Machine learning method: XGBoost

XGBoost^32^ has demonstrated excellence in various fields, including civil infrastructure management^33–35^ and flood risk management^36,37^. Its ability to handle large datasets and capture intricate relationships enables accurate predictions and informed decision-making^37^. The robustness and efficiency of XGBoost make it suitable for real-time applications, providing valuable insights for disaster preparedness and response.

In this study, we selected XGBoost over other traditional machine learning models (support vector machine, random forest, decision tree) and deep learning models (multi-layer perceptron). XGBoost produced better results on the initial formulation of the problem, i.e., predicting flood depth after training a generic cell based on precipitation, topography, and proximity to channel. After further experiments, we found that a separate model for each cell was more successful than a single, universal model applicable to any cell of the study area. Even though the similarity of precipitations on two contiguous cells might advise against training independent models, this choice allowed us to better control for the temporal and spatial dependencies of precipitation. To train each model effectively, we first implemented 10-fold cross-validation on XGBoost regressor, and then ran the hyperparameter tuning by random search, seeking the optimal parameter set based on the highest Area Under the Receiver Operating Characteristics (ROC-AUC) metrics. The objective function was to minimize squared errors, with a learning rate of 0.01. The XGBoost model constructed 1000 trees with a maximum depth of 5 to balance complexity and performance. L1 regularization was applied to prevent overfitting, whereas additional randomness (subsample ratio of columns set to 0.3) introduced non-linearity and ensured robustness. Once the optimal hyperparameter was set, 592 storm events modeled with HEC-RAS 2D were partitioned into training (356 events), validation (118 events), and test (118 events) datasets. In addition, all features were normalized on a 0-1 range using a constant feature scale derived from the training data.

The study involved two experiment setups. The first incorporated peak and cumulative precipitation within each cell. The second setup added watershed heavy cumulative precipitation ratio and heavy peak precipitation ratios, providing information on rainfall intensity and spatial pattern. As part of its routine, XGBoost computes feature importance scores, which measure the frequency with which a feature is used to split data across trees in the model^38^. In practical terms, feature importance scores assess the contribution and impact of individual features on the model’s prediction (Fig. 4). The XGBoost models were trained on 20 CPUs for parallel computation, with each experiment taking approximately two hours. The model checkpoint was stored at the optimal validation RMSE for each cell and used for flood depth prediction in the case study.

### Supplementary Information

Supplementary Information.

## Data Availability

The data that support the findings of this study is available from the corresponding author upon request.

## Code availability

The code that supports the findings of this study is available from the corresponding author upon request.
