# Supplementary figures and images for: Predicting peak inundation depths with a physics informed machine learning model
Source: Sci Rep. 2024 Jun 27;14:14826. doi: 10.1038/s41598-024-65570-8 (PMC11211320; doi:10.1038/s41598-024-65570-8)

## Supplemental Material

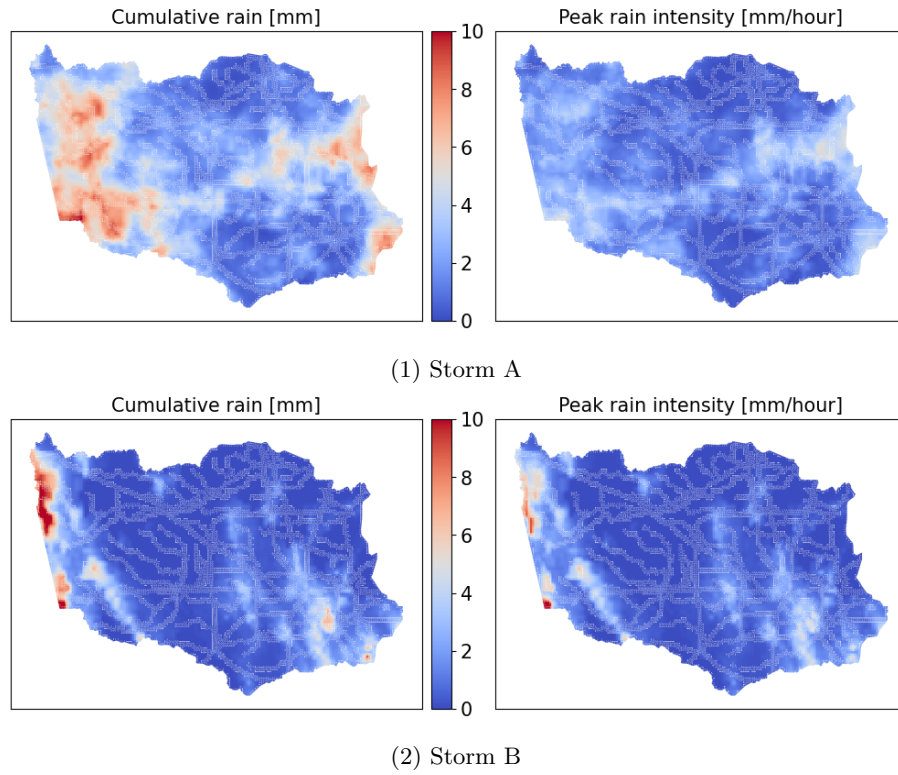

Figure A: Cumulative and peak rainfall maps of different storm events.

Supplement: Supplementary file 1 — Supplementary Information. [file 41598_2024_65570_MOESM1_ESM.pdf]
